# Cytokine Dysregulation in *MECP2*- and *CDKL5*-Related Rett Syndrome: Relationships with Aberrant Redox Homeostasis, Inflammation, and *ω*-3 PUFAs

**DOI:** 10.1155/2015/421624

**Published:** 2015-07-08

**Authors:** Silvia Leoncini, Claudio De Felice, Cinzia Signorini, Gloria Zollo, Alessio Cortelazzo, Thierry Durand, Jean-Marie Galano, Roberto Guerranti, Marcello Rossi, Lucia Ciccoli, Joussef Hayek

**Affiliations:** ^1^Child Neuropsychiatry Unit, University Hospital, Azienda Ospedaliera Universitaria Senese (AOUS), Policlinico “S. M. alle Scotte”, 53100 Siena, Italy; ^2^Department of Molecular and Developmental Medicine, University of Siena, 53100 Siena, Italy; ^3^Neonatal Intensive Care Unit, University Hospital, AOUS, Policlinico “S. M. alle Scotte”, 53100 Siena, Italy; ^4^Department of Medical Biotechnologies, University of Siena, 53100 Siena, Italy; ^5^Institut des Biomolécules Max Mousseron (IBMM), UMR 5247, CNRS/UM/ENSCM, BP 14491, 34093 Montpellier Cedex 5, France; ^6^Respiratory Pathophysiology and Rehabilitation Unit, University Hospital, AOUS, Policlinico “S. M. alle Scotte”, 53100 Siena, Italy

## Abstract

An involvement of the immune system has been suggested in Rett syndrome (RTT), a devastating neurodevelopmental disorder related to oxidative stress, and caused by a mutation in the methyl-CpG binding protein 2 gene (*MECP2*) or, more rarely, cyclin-dependent kinase-like 5 (*CDKL5*). To date, it is unclear whether both mutations may have an impact on the circulating cytokine patterns. In the present study, cytokines involved in the Th1-, Th2-, and T regulatory (T-reg) response, as well as chemokines, were investigated in *MECP2*- (*MECP2*-RTT) (*n* = 16) and *CDKL5*-Rett syndrome (*CDKL5*-RTT) (*n* = 8), before and after *ω*-3 polyunsaturated fatty acids (PUFAs) supplementation. A major cytokine dysregulation was evidenced in untreated RTT patients. In *MECP2*-RTT, a Th2-shifted balance was evidenced, whereas in *CDKL5*-RTT both Th1- and Th2-related cytokines (except for IL-4) were upregulated. In *MECP2*-RTT, decreased levels of IL-22 were observed, whereas increased IL-22 and T-reg cytokine levels were evidenced in *CDKL5*-RTT. Chemokines were unchanged. The cytokine dysregulation was proportional to clinical severity, inflammatory status, and redox imbalance. Omega-3 PUFAs partially counterbalanced cytokine changes, as well as aberrant redox homeostasis and the inflammatory status. RTT is associated with a subclinical immune dysregulation as the likely consequence of a defective inflammation regulatory signaling system.

## 1. Introduction

Rett syndrome (RTT) (OMIM ID: 312750) is a severe progressive neurological disorder usually linked to two major X-linked gene mutations, that is, methyl-CpG binding protein 2 gene (*MECP2*) and cyclin-dependent kinase-like 5 (*CDKL5*) [1–3].

*MECP2* mutations are known to cause up to 90–95% of cases with typical clinical presentation (*MECP2*-RTT) which include early neurological regression followed by loss of acquired cognitive, social, and motor skills in a typical 4-stage neurological regression, together with development of autistic behavior [4]. The typical disease, manifesting above 6–18 months almost exclusively in girls, represents the second most common cause of severe intellective disorder in the female gender [1] and its frequency is approximatively 1 : 10,000 with a random worldwide distribution [5].* MECP2* mutations have also been reported in a milder syndrome phenotype (i.e., preserved speech variant), as well as other non-RTT disorders, such as Angelman syndrome, mild learning disability in females, neonatal encephalopathy (largely predominant in males), and X-linked intellectual disability [6].

Although MeCP2 has been extensively studied in the central nervous system [7], MeCP2 is a ubiquitous nuclear protein [8]. However, the molecular mechanisms by which MeCP2 deficiency drives pathology in RTT remain elusive. MeCP2 is currently considered a multifunctional regulator of gene transcription, involved in RNA splicing and chromatin remodelling [7]. A close biochemical and genetic relationship exists between MeCP2 and CDKL5 in that the encoded CDKL5 protein is known to be a kinase able to mediate MeCP2 phosphorylation [3] and that CDKL5 has been reported to be a MeCP2-repressed target gene [9].* CDKL5*-related RTT (*CDKL5*-RTT) is known as the early-onset seizures variant (ESV) and is associated with severe early-onset seizures and partially different clinical RTT-like features [10], so that recently the CDKL5 disorder has been proposed as an independent clinical entity rather than an additional RTT variant [11].

Our prior studies indicate that (1) MeCP2 is involved—either directly or indirectly—in the regulation of the redox balance in RTT [12–18]; (2) a direct evidence of the link between MeCP2 deficiency, oxidative stress (OS), and RTT pathology exists in murine models of the disease [18]; (3) a redox imbalance has also been evidenced in* CDKL5*-RTT [16]. Proofs for a direct relationship between the MeCP2 protein and OS are lacking to date. However, we have previously demonstrated that OS in brains of mouse with* Mecp2* loss-of-function mutations precedes and accompanies symptoms of the disease and is rescued by selective Mecp2 gene reexpression [18]. Whether mitochondria are the main, or the only, major intracellular source for abnormal redox status in RTT [19–23] or other “*MECP2*-pathies” is still to be ascertained, given that no major morphological alterations in mitochondria have been evidenced by our group at the transmission electron microscopy of primary cultures of skin fibroblasts from* MECP2*-RTT, whereas major changes were observed in the endoplasmic reticulum, along with cytoplasmic multilamellar bodies [17].

*MECP2*-RTT appears to be intimately related to inflammation [24, 25]. There is prior evidence that MeCP2 associates with CpG elements of the regulatory regions of* Foxp3* gene, a known transcription factor needed for the generation of T regulatory (T-reg) cells [26]. This observation supports the concept that MeCP2 could play a regulatory role in T cells' resilience to inflammation [27]. Furthermore, a very recent report demonstrates that MeCP2 acts as a regulator of the response to inflammatory stimuli in the microglia and macrophages of* Mecp2*-null mice [28].

While an impact on the immune system in gain-of-function mutations, such as the* MECP2* duplication syndrome is evidenced [29, 30], no clinically relevant primary immune deficiency has been previously reported in either* MECP2*-RTT or* CDKL5*-RTT. Nevertheless, an involvement of the immune system in* MECP2*-RTT has been previously suggested [31–35] although conflicting negative evidence also exists [36–38].

Preliminary findings by Ariani suggest that a reduced frequency of HLA-B39 is present in* MECP2*-RTT [39], whose reasons and biological consequences need to be clarified. Some disrupting mutations in chemokines and chemokines receptors have been reported by Grillo et al. in the preserved speech variant of RTT, thus suggesting this combination of mutations along with the* MECP2* loss-of-function may modulate the immune system [40].

An association between* MECP2* gene polymorphisms and autoimmune disease, such as systemic lupus erythematosus (LES) [41, 42] and primary Sjögren's syndrome [43], has been reported. High levels of serum anti-N (Glc) autoantibodies of the IgM fraction have been recently reported in RTT [44]. A direct relationship between MeCP2 and the immune system has been recently evidenced, since MeCP2 has been found to be indispensable for the differentiation of naïve CD4+ T cells into Th17 cells and for the commitment of naïve CD4+ T cells to the Th1 lineage [30]. Moreover, MeCP2 has been shown to play a critical role in promoting multiple cytokine-mediated signaling pathway through a MeCP2-miR-124-suppressor of cytokine signaling 5 (SOCS5) axis known to be indispensable for the activation of signal transducer and activator of transcription 3 (STAT3) and STAT1 in CD4+ T cells [30]. This signaling pathway appears to be indispensable for the activation of signal transducer and activator of transcription (i.e., STAT3) in naïve CD4+ T cells, with consequent generation of Th17 cells [30].

In the present study, we evaluated systemic cytokine patterns, oxidative stress markers, and inflammatory status in both typical* MECP2*-RTT and* CDKL5*-RTT as a function of a 12-month supplementation with *ω*-3 polyunsaturated fatty acids (PUFAs).

## 2. Methods

### 2.1. Subjects

The study included 24 Rett female patients with different clinical diagnosis: typical Rett syndrome (*n* = 16, mean age: 14.2 ± 9.5 years, range: 3–39) with demonstrated* MECP2* gene mutation and ESV (*n* = 8, mean age: 8.7 ± 4.8 years, range: 2–17) with demonstrated* CDKL5* gene mutation. Rett syndrome diagnosis and inclusion/exclusion criteria were based on the recently revised Rett syndrome nomenclature consensus [45].

Clinical examinations and blood samplings in the* MECP2*- and* CDKL5*-RTT patients' groups were performed before and after *ω*-3 polyunsaturated fatty acids (PUFAs) supplementation during the routine follow-up study at hospital admission. Given the specific aims of this study, patients with clinically evident inflammatory conditions (i.e., upper respiratory tract infection, pneumonia, urinary infection, stomatitis, and periodontal inflammation), either acute or chronic, were excluded.

None of the patients at the time of enrollment were on anti-inflammatory drugs or undergoing supplementation with other known antioxidants. All the patients were consecutively admitted to the “Rett Syndrome National Reference Centre” (Head Professor J.H.) of the University Hospital of the Azienda Ospedaliera Universitaria Senese (AOUS). The subjects examined in this study were on a typical Mediterranean diet. Rett syndrome clinical severity was assessed using either the total clinical severity score (CSS), a validated clinical rating specifically designed for RTT, or the “compressed” CSS, both based on 13 individual ordinal categories measuring clinical features common in RTT [46]. Healthy female control subjects who have comparable age (*n* = 24; age: 13.4 ± 4.8 years, range: 2–32) were also included. Blood samplings from the control group were carried out during routine health checks, sports, blood donations, or periodic clinical checks.

The study was conducted with the approval of the Institutional Review Board of the AOUS. All informed consents were obtained from either the parents or the legal tutors of the enrolled patients or directly from healthy adults.

### 2.2. Omega-3 PUFAs Oral Supplementation

All* MECP2*- and* CDKL5*-related RTT patients were supplemented with *ω*-3 PUFAs oil for 12 months. Administered *ω*-3 PUFAs were in the form of fish oil before food intake (Norwegian Fish Oil AS, Trondheim, Norway, product number HO320-6; Italian importer: Transforma AS Italia, Forlì, Italy; Italian Ministry registration code: 10 43863-Y) at a dose of 5 mL twice daily, corresponding to docosahexaenoic acid (DHA, 22 : 6 *ω*-3) 74.3 ± 6.8 mg/kg b.w./day and eicosapentaenoic acid (EPA, 20 : 5 *ω*-3) 119.7 ± 10.6 mg/kg b.w./day, with a total *ω*-3 PUFAs of 248.2 ± 25.1 mg/kg b.w./day. Use of EPA plus DHA in RTT was approved by the AOUS Ethical Committee. All data obtained in* MECP2*- and* CDKL5*-related RTT patients after *ω*-3 PUFAs supplementation were compared to those obtained before the supplementation.

### 2.3. Inflammatory Marker

We measured erythrocyte sedimentation rate (ESR) as nonspecific marker of inflammation. ESR was assayed on a widely used automated system (i.e., “TEST 1”) measuring the aggregation capacity of red blood cells (RBCs) by an infrared ray microphotometer with a light wavelength of 950 nm [47].

### 2.4. Sample Collection

All samplings from RTT patients and healthy controls were carried out around 8 AM after overnight fasting. Blood was collected in heparinized tubes and used for cytokine evaluations and oxidative stress (OS) markers determinations. In particular, blood samples were centrifuged at 2,400 g for 15 min at room temperature. The platelet poor plasma was saved, and the buffy coat was removed by aspiration. RBCs were washed twice with physiological solution, resuspended in Ringer solution (125 mM NaCl, 5 mM KCl, 1 mM MgSO_4_, 32 mM HEPES, 5 mM glucose, and 1 mM CaCl_2_), pH 7.4 as a 50% (vol/vol) suspension, and then used for the determination of intraerythrocyte non-protein-bound iron (IE-NPBI). Plasma was used for cytokine, and OS marker (F_2_-isoprostanes: F_2_-IsoPs; F_2_-dihomo-isoprostanes: F_2_-dihomo-IsoPs; F_4_-neuroprostanes: F_4_-NeuroPs, and plasma non-protein-bound iron: P-NPBI) determinations. All manipulations were carried out within 2 h after sample collection. For all isoprostane determinations, butylated hydroxytoluene (90 *μ*M) was added to plasma as an antioxidant and stored under nitrogen at −70°C until analysis.

For serum preparation (IFN-*γ* and TGF-*β*1 evaluations), blood samples were left to coagulate for 15–30 min at RT and centrifuged at RT for 10 minutes at 2000 g. Separated sera were kept in aliquots at −80°C until the time of assay.

### 2.5. Enzyme-Linked Immunosorbent Assay (ELISA) for Cytokines

After blood collection, plasma was separated as soon as possible after centrifugation to avoid TNF-*α* production by blood cells that falsely could increase its values [48]. Levels of T helper type 1- (Th1-) related cytokine response (IL-1*β*, TNF-*α*, IFN-*γ*, and IL-12p70), T helper type 2- (Th2-) related cytokine response (IL-4, IL-5, IL-6, IL-10, IL-13, and IL-33), proinflammatory cytokines IL-17A and IL-22, regulatory T (T-reg) cytokine (TGF-*β*1), and chemokines (IL-8/CXCL8, IP-10/CXCL10, I-TAC/CXCL11, and RANTES/CCL5) were quantified in plasma or serum samples from the examined groups by enzyme-linked immunosorbent assay (ELISA), carried out in accordance with the manufacturer's instructions (Quantikine, R & D Systems, Minneapolis, MN, USA). Sera samples were used for IFN-*γ* and TGF-*β*1 determinations. The minimum detectable dose (MDD) was 1 pg/mL for IL-1*β*, 10 pg/mL for IL-4, 0.29 pg/mL for IL-5, 0.70 pg/mL for IL-6, 3.5 pg/mL for IL-8, 3.9 pg/mL for IL-10, 5 pg/mL for IL-12p70, 13.2 pg/mL for IL-13, 15 pg/mL for IL-17A, 2.7 pg/mL for IL-22, 0.519 pg/mL for IL-33, 1.67 pg/mL for IP-10, 13.9 pg/mL for I-TAC, 2.0 pg/mL for RANTES, and 1.6 pg/mL for TNF-*α*. MDD for IFN-*γ* and TGF-*β*1 was 8.0 pg/mL and 4.61 pg/mL, respectively. The levels of plasma IL-37, an anti-inflammatory cytokine recently identified [49], were determined using a commercially available ELISA kit (AdipoGen, Switzerland), according to the manufacturers' instruction. MDD for IL-37 was 10 pg/mL. The samples were performed in duplicate to guarantee the reproducibility of the kits. For reference values, we referred to the reference range as derived from previous reports or, in the lack of prior published results, manufacturer's data sheets. IL-10 is considered as both Th2 cytokine and T-reg cytokine [50].

Th2/Th1 ratio was defined based on the ratio of IL-4, IL-5, IL-6, or IL-10 Th2-related cytokines and proinflammatory IFN-*γ* and TNF-*α* Th1-related cytokines [51].

### 2.6. Redox Status Evaluations

We evaluated the following markers: intraerythrocyte and plasma non-protein-bound iron (IE-NPBI and P-NPBI) (as prooxidant factors) [12], F_2_-IsoPs (as markers of systemic lipoperoxidation) [12], F_2_-dihomo-IsoPs (as markers of glia membrane damage/brain white matter) [15], F_4_-NeuroPs (as markers of neuronal membrane damage/brain gray matter) [14], and blood GSH/GSSG ratio as indicator of antioxidant defence [52].

In particular, P-NPBI and IE-NPBI were determined as a desferrioxamine-iron complex by high-performance liquid chromatography (HPLC) [12]. F_2_-IsoPs, F_2_-dihomo-IsoPs and F_4_-NeuroPs (end-oxidation products of arachidonic acid, adrenic acid, and docosahexaenoic acid, resp.) were determined by a gas chromatography/negative ion chemical ionization tandem mass spectrometry (GC/NICI-MS/MS) analysis after solid phase extraction and derivatization steps. For F_2_-IsoPs, F_2_-dihomo-IsoPs, and F_4_-NeuroPs the measured ions were the product ions at *m*/*z* 299, 327, and 323, respectively [12–15].

Blood GSH and GSSG levels were determined by an enzymatic recycling procedure according to Tietze et al. [53] and Baker et al. [54].

### 2.7. Data Analysis

All variables were tested for normal distribution (D'Agostino-Pearson test) and data were presented as mean and standard deviation unless otherwise specified. Statistical analysis for circulating cytokine levels in the groups at baseline and after *ω*-3 PUFAs supplementation was carried out using Student's *t*-test and one-way ANOVA test. Bonferroni-corrected significance levels were used for multiple *t*-tests. Associations between variables were tested by univariate linear regression analysis and multiple regression analyses. A two-sided *P* < 0.05 was considered to indicate statistical significance. The MedCalc version 12.1.4 statistical software package (MedCalc Software, Mariakerke, Belgium) was used.

## 3. Results

### 3.1. Clinical Severity in* MECP2*- and* CDKL5*-RTT

In the untreated RTT population, mean CCSS for* MECP2*-RTT and* CDKL5*-RTT was 7.1 ± 0.6 (values range 5–11) and 4.0 ± 0.5 (values range 7-8), respectively. Major clinical features included neurological regression history (100%), somatic growth deficiency (31.2%* MECP2 versus* 50%* CDKL5*), microcephaly (100%), loss of spontaneous ambulation (43.7%* MECP2 versus* 87.5%* CDKL5*), loss of purposeful hand use (87.5%), scoliosis (37.5%* MECP2*-mutated patients), absent verbal language (93.7%* MECP2 versus* 100%* CDKL5*), absent nonverbal communication (6.2%* MECP2 versus* 0%* CDKL5*), respiratory dysfunction (43.7%* MECP2 versus* 12.5%* CDKL5*), autonomic nervous system signs (62.5%), stereotypies (100%), and epilepsy (43.7%* MECP2 versus* 100%* CDKL5*).

### 3.2. Basal Cytokine Patterns in* MECP2*- and* CDKL5*-RTT

#### 3.2.1. *MECP2*- RTT

**Figure 1:**
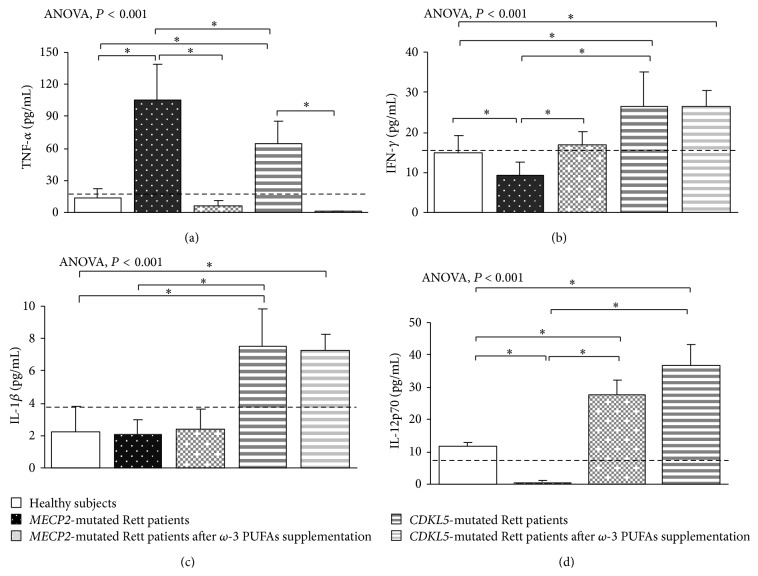
Circulating Th1-related cytokines in* MECP2*- and* CDKL5*-mutated Rett patients before and after *ω*-3 PUFAs supplementation. Data are expressed as means ± standard deviations. Asterisks denote significant* post hoc* pairwise tests (*P* < 0.05). The dashed line denotes upper range limit as obtained from manufacturer's data sheet.

**Figure 2:**
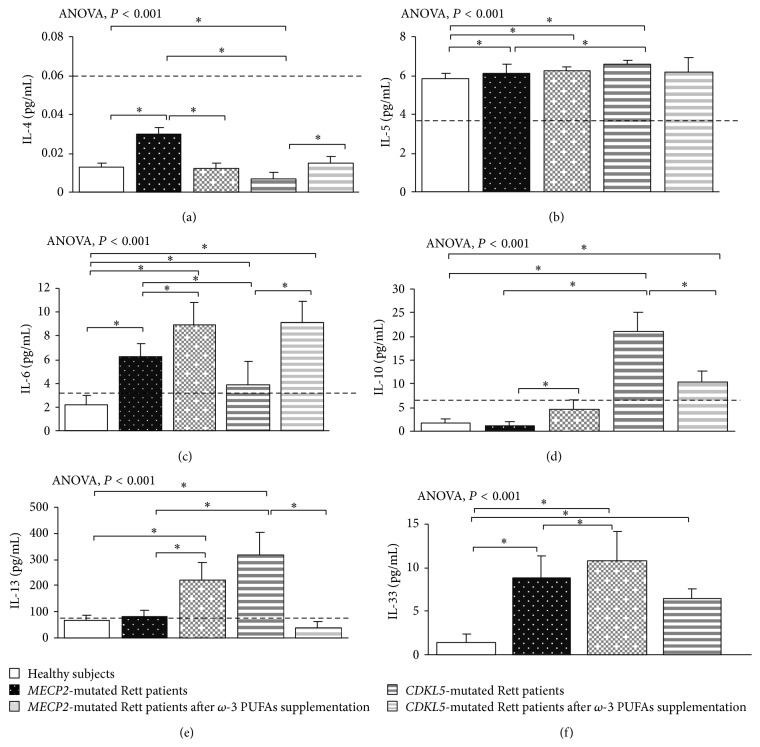
Circulating Th2-related cytokines in* MECP2*- and* CDKL5*-mutated Rett patients before and after *ω*-3 PUFAs supplementation. Data are expressed as means ± standard deviations. Asterisks denote significant* post hoc* pairwise tests (*P* < 0.05). The dashed line denotes upper range limit as obtained from manufacturer's data sheet or from published literature. For IL-4, normal range is referred to literature [55]. For IL-13, normal range is referred to literature [56]. For IL-33, normal range is not indicated in the manufacturer's data sheet. However, some authors reported also a range [57].

**Figure 3:**
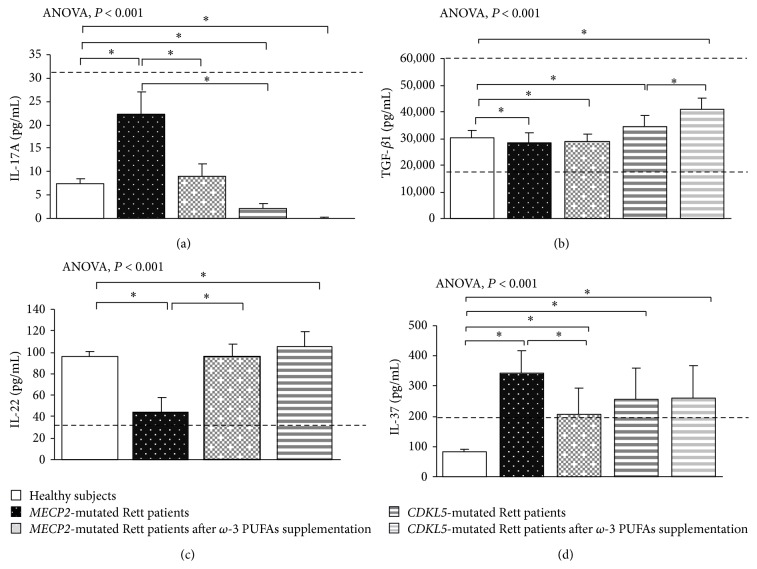
Circulating IL-17A, IL-22, TGF-*β*1, and IL-37 in* MECP2*- and* CDKL5*-mutated Rett patients before and after *ω*-3 PUFAs supplementation. Data are expressed as means ± standard deviations. Asterisks denote significant* post hoc* pairwise tests (*P* < 0.05). Note: levels of IL-10 are shown in Figure 2 since IL-10 is considered as both Th2 cytokine and T-reg cytokine. The dashed line denotes upper range limit as obtained from manufacturer's data sheet or from published literature. For TGF-*β*1, normal range is indicated by dashed lines (upper and lower range limits). For IL-37, normal range is referred to in literature [58].

**Figure 4:**
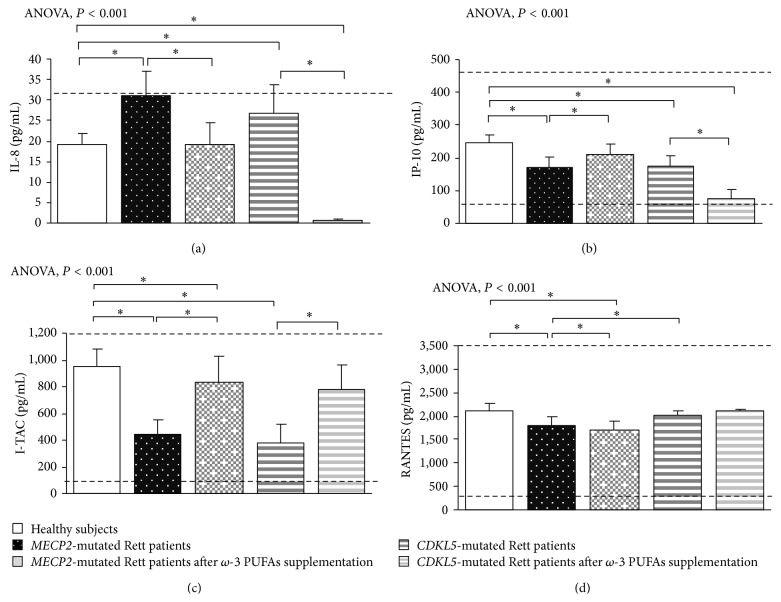
Circulating chemokines in* MECP2*- and* CDKL5*-mutated Rett patients before and after *ω*-3 PUFAs supplementation. Data are expressed as means ± standard deviations. Asterisks denote significant* post hoc* pairwise tests (*P* < 0.05). The dashed line denotes upper and lower range limits as obtained from manufacturer's data sheet. For IP-10, I-TAC, and RANTES, normal range is indicated by dashed lines.

In typical* MECP2*-RTT, increased levels of TNF-*α*, IL-5, IL-6, and IL-37 plasma levels (*P* < 0.05) and a slight increase in IL-8 were evidenced as compared to reference range, whereas decreased levels of IFN-*γ*, IL-12p70, and IL-22 were observed (*P* < 0.05). No changes for IL-1*β*, IL-4, IL-10, IL-13, IL-17A, TGF-*β*1, IP-10, I-TAC, and RANTES were evidenced as compared to published range (Figures 1, 2, 3, and 4) [55–58].

Nevertheless, when compared to the examined healthy control group, typical* MECP2*-RTT patients also showed increased levels of IL-4, IL-8, IL-17A, and IL-33 (*P* < 0.05) and decreased TGF-*β*1, IP-10, I-TAC, and RANTES levels (*P* < 0.05). No changes for IL-1*β*, IL-10, and IL-13 were evidenced.

**Figure 5:**
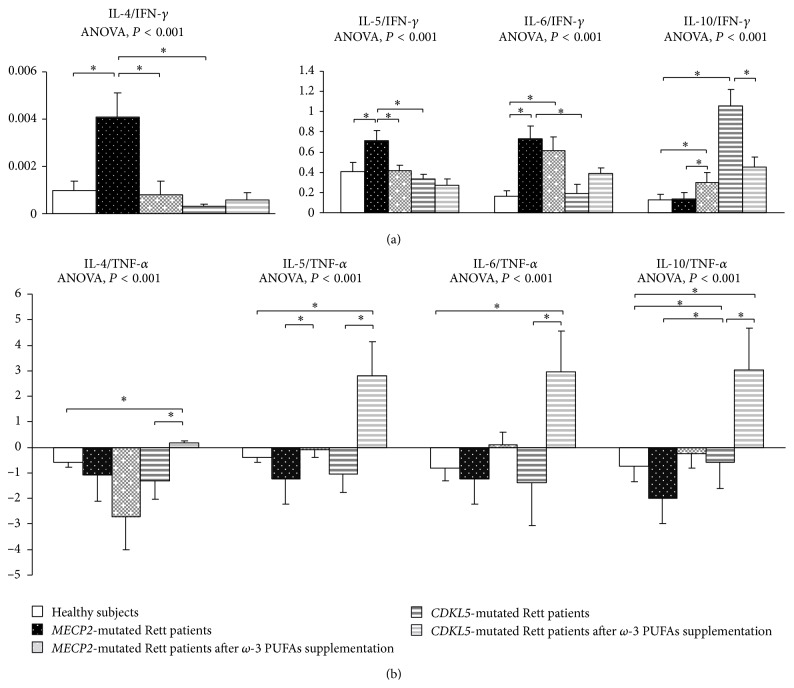
Th2/Th1 ratio in* MECP2*- and* CDKL5*-mutated Rett patients before and after *ω*-3 PUFAs supplementation. (a) Comparative Th2/Th1 ratios defined on the ratio of Th2-related cytokines (IL-4, IL-5, IL-6, and IL-10) and INF-*γ*. Data are expressed as means ± standard deviations. Asterisks denote significant* post hoc* pairwise tests (*P* < 0.05). (b) Comparative Th2/Th1 ratios defined on the ratio of Th2-related cytokines (IL-4, IL-5, IL-6, and IL-10) and TNF-*α*. Th2/Th1 ratio values are expressed in logarithmic scale and indicated as means ± standard deviations. Asterisks denote significant* post hoc* pairwise tests (*P* < 0.05).

When compared to control population and based on IFN-*γ*,* MECP2*-RTT showed a Th2 shift (i.e., increased IL-4/IFN-*γ*, IL-5/IFN-*γ*, and IL-6/IFN-*γ* ratios) (*P* < 0.05) (Figure 5). Ratios based on TNF-*α* were difficult to interpret given the bulk increase of TNF-*α* level.

Overall,* MECP2*-RTT showed decreased levels of Th1-related cytokines (i.e., IFN-*γ*, IL-12p70), with the remarkable exception of increased TNF-*α* levels. Altogether, a Th2 shift (increased IL-5 and IL-6) was detectable.

In addition, increased levels of the anti-inflammatory cytokine IL-37 and decreased IL-22 concentration were also observed. No significant changes in T-reg-related cytokines (i.e., IL-10 and TGF- *β*1) and chemokines were present.

#### 3.2.2. *CDKL5*-RTT

In* CDKL5*-RTT, increased levels of TNF-*α*, IFN-*γ*, IL-1*β*, IL-12p70, IL-5, IL-6, IL-10, IL-13, TGF-*β*1, IL-22, and IL-37 (*P* < 0.05) were evidenced. No changes were observed for all the other examined cytokines (Figures 1, 2, 3, and 4) [55–58].

When compared to our control group,* CDKL5*-RTT also showed increased levels of IL-8, IL-33 (*P* < 0.05) with decreased IL-4, IL-17A, IP-10, and I-TAC levels (*P* < 0.05). No changes were evidenced for RANTES. Apart from increased IL-10/IFN-*γ* and IL-10/TNF-*α* ratios (i.e., IL-10 is a cytokine classified as both a Th2 cytokine and T-reg-related cytokine), no changes were observed for all the other examined ratios (Figure 5).

Overall, both Th1- and Th2-related (except for IL-4) cytokines were upregulated in* CDKL5*-RTT, together with increased levels of IL-22 and T-reg-related cytokines. No changes were observed for chemokine levels.

#### 3.2.3. Relative Changes (Fold Variations)

In order to account for real changes, that is, independently of absolute basal concentrations, values data were submitted to double standardization (i.e., basal values of untreated* MECP2*- and* CDKL5*-RTT patients* versus* control values and treated* MECP2*- and* CDKL5*-RTT patients* versus* untreated). After standardization, some differences lost weight, whereas others gained statistical significance (Supplementary Tables  1 and  2 in the Supplementary Material available online at http://dx.doi.org/10.1155/2015/421624). In particular, changes in IL-10 in* MECP2*-RTT and IL-6, TGF-*β*1, IP-10, IL-5/TNF-*α* ratio, IL-6/TNF-*α* ratio, and IL-10/TNF-*α* ratio in CDKL5-RTT were found to be not significant after *ω*-3 PUFAs treatment, whereas changes in IL-5, IL-6/IFN-*γ* ratio, IL-4/TNF-*α* ratio, and IL-6/TNF-*α* ratio in* MECP2*-RTT and IFN-*γ*, IL-1*β*, IL-5, IL-37, and IL-4/IFN-*γ* ratio, F_4_-NeuroPs, and GSH/GSSG ratio in* CDKL5*-RTT were found to be statistically significant.

#### 3.2.4. Summary Effects of Circulating Basal Cytokine Changes in* MECP2*- and* CDKL5*-RTT

**Figure 6:**
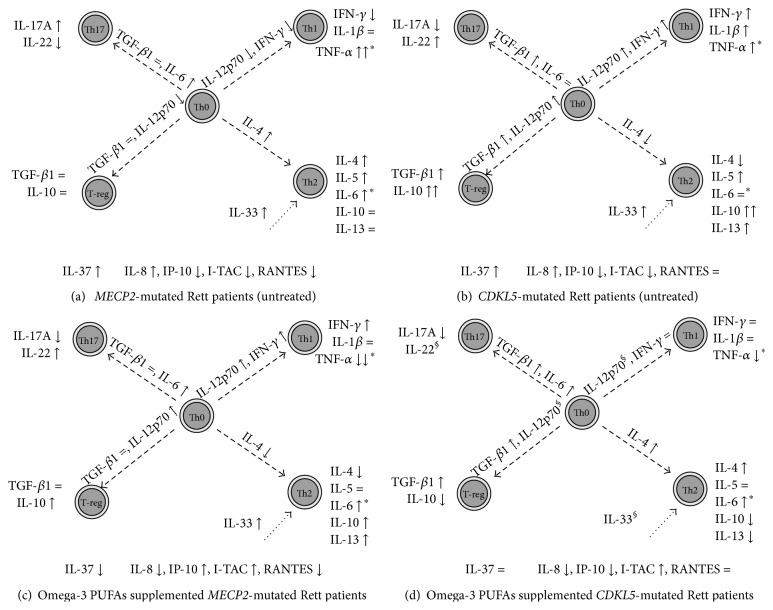
Schematic cytokine changes in* MECP2*- and* CDKL5*-RTT. Data were based on comparisons between* MECP2*- and* CDKL5*-related Rett patients and healthy subjects reported in Figures 1, 2, 3, and 4. (a) and (b) The symbol ↓, ↑, or = indicates a significant decrease, increase, or unchanged value of the examined cytokine* versus* examined healthy subject values. (c) and (d) The symbol ↓, ↑, or = indicates a significant decrease, increase, or unchanged value of the examined cytokine* versus* untreated* MECP2*- and* CDKL5*-mutated Rett patients values. ^*∗*^Cytokines mainly secreted by macrophages; ^§^cytokines whose assay is not available in the *ω*-3 PUFAs supplemented* CDKL5*-RTT.

In typical* MECP2*-RTT, the Th2 and Th17 lineages appear to be activated. In particular, activation of Th2 lineage is more evident as compared to that of Th17 line. The Th1 lineage appears to be inhibited with the single exception of TNF-*α*, while no changes were observed regarding the T-reg lineage. Besides, chemokines appear to be inhibited with the single exception of IL-8. IL-33, known to be involved in Th2 differentiation, is increased. IL-37, known to counteract inflammatory status, appears to be increased and likely reflects a proinflammatory status in the untreated* MECP2*-RTT population (Figure 6(a)). In* CDKL5*-RTT, the Th1 and T-reg lineages appear to be strongly activated, whereas paradoxical effects were observed regarding Th17. An important feature of untreated* CDKL5*-RTT appears to be a strongly increased production of IL-10 dominating the Th2 pattern changes. Chemokines and IL-37 show the same pattern observed in* MECP2*-RTT (Figure 6(b)).

Overall, several of the observed cytokine pattern changes in untreated RTT (either* MECP2*- or* CDKL5*-related RTT patients) appear to reflect a likely macrophage dysregulation/dysfunction as suggested by Cronk et al. [28]. In particular, in* MECP2*-RTT, a strongly increased production of TNF-*α* was evidenced, whereas apart from TNF-*α* a strongly increased release of IL-10 and IL-12p70 was observed in* CDKL5*-RTT.

### 3.3. Redox Status in* MECP2*- and* CDKL5*-RTT

**Figure 7:**
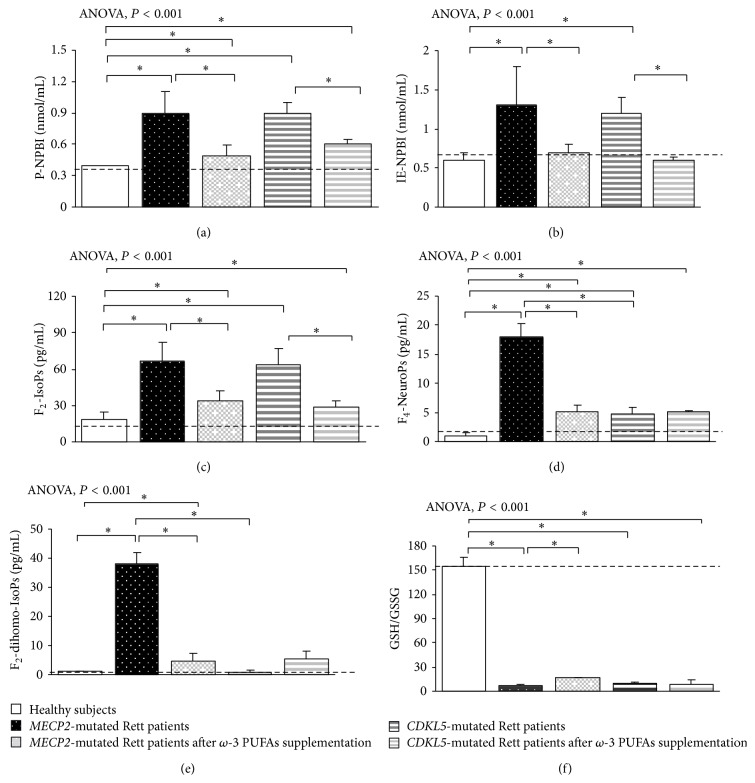
Systemic redox status in* MECP2*- and* CDKL5*-mutated Rett patients before and after *ω*-3 PUFAs supplementation. Data are expressed as means ± standard deviations. Asterisks denote significant* post hoc* pairwise tests (*P* < 0.05). P-NPBI: plasma non-protein-bound iron; F_2_-IsoPs: plasma F_2_-isoprostanes; F_2_-dihomo-IsoPs: plasma F_2_-dihomo-isoprostanes; F_4_-NeuroPs: plasma F_4_-neuroprostanes; GSH: reduced glutathione; GSSG: oxidized glutathione. The dashed line denotes upper range limit from laboratory normal reference values.

In typical* MECP2*-RTT an oxidative imbalance was evidenced, thus confirming our prior observations [12–15]. In particular, a significant increase in P-NPBI and IE-NPBI (i.e., free redox active iron), F_2_-IsoPs (i.e., markers of systemic OS), F_4_-NeuroPs (i.e., markers of brain grey matter oxidative injury), and F_2_-dihomo-IsoPs (i.e., markers of brain white matter oxidative injury) levels (all *P* < 0.05) as compared to healthy subjects is observed. In* CDKL5*-RTT, similar results were found (Figure 7), confirming our preliminary observations [16]. However, some differences between* MECP2*-RTT and* CDKL5*-RTT were observed. While all the examined OS markers were found to be increased in* MECP2*-RTT, F_2_-dihomo-IsoPs plasma levels in* CDKL5*-RTT were found to be comparable to those of the control group. The observed redox imbalance was mirrored by a strongly reduced GSH/GSSG ratio as detectable in both conditions (Figure 7).

### 3.4. Inflammatory Status in* MECP2*- and* CDKL5*-RTT

**Figure 8:**
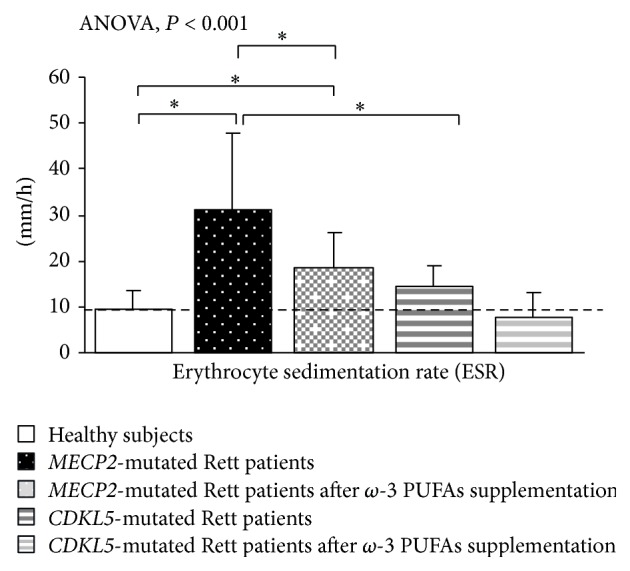
Inflammatory status in* MECP2*- and* CDKL5*-mutated Rett patients before and after *ω*-3 PUFAs supplementation. Data are expressed as means ± standard deviations. Asterisks denote significant* post hoc* pairwise tests (*P* < 0.05). The dashed line denotes upper range limit as obtained from clinical laboratory.

Elevated ESR values were detectable in* MECP2*-RTT as compared to healthy subjects (mean value 9.4 ± 5.8 mm/h), thus confirming the presence of a subclinical inflammatory status in* MECP2*-RTT patients [24]. A slight, albeit not statistically significant, increase in ESR was observed in* CDKL5*-RTT patients (Figure 8).

### 3.5. Correlations between Cytokines, Oxidative Stress, Inflammatory Status, and Phenotype Severity

In the RTT population, the cytokine-dependent response was significantly correlated with redox imbalance (i.e., P-NPBI, IE-NPBI, F_2_-IsoPs, F_4_-NeuroPs, F_2_-dihomo-IsoPs, and GSH/GSSG ratio), inflammatory index (i.e., ESR), or compressed clinical severity score (i.e., CCSS). Relatively to the different cytokines, in Table 1 are reported the correlation coefficients. Interestingly, we evidenced significant correlations between circulating cytokine levels, OS markers, and some individual clinical features common in RTT syndrome (i.e., seizures and scoliosis). The presence of scoliosis was significantly associated with IL-4 (*r* = 0.424, *P* < 0.0001), IFN-*γ* (*r* = −0.458, *P* < 0.0001), IL-12p70 (*r* = −0.327, *P* = 0.0029), IL-4/IFN-*γ* ratio (*r* = 0.491, *P* < 0.0001), IL-5/IFN-*γ* ratio (*r* = 0.442, *P* = 0.0001), and IL-6/IFN-*γ* ratio (*r* = 0.429, *P* = 0.0001). Epilepsy was associated with IL-5 (*r* = 0.347, *P* = 0.0020), IL-10 (*r* = 0.328, *P* = 0.0036), IL-12p70 (*r* = 0.370, *P* = 0.0001), IL-13 (*r* = 0.346, *P* = 0.0021), and F_2_-IsoPs (*r* = 0.536, *P* < 0.0001).

A stepwise multiple regression analysis showed that clinical severity (i.e., CCSS) was significantly associated with cytokine dysregulation and aberrant redox homeostasis (*P* < 0.001) (Table 2). In fact, circulating cytokine levels accounted for 40.7% to 81.2% of the whole variance observed for inflammation and redox imbalance. In particular, CCSS was found to be related to TNF-*α*, IL-17A, IL-4, and IL-12p70 levels and associated with F_2_-IsoPs, F_4_-NeuroPs, and GSH/GSSG ratio. On the other hand, OS markers were mainly related to TNF-*α*, IL-37, and I-TAC levels. This data suggests the existence of a complex interplay between cytokine modulation, redox imbalance, inflammatory status, and clinical severity in RTT.

### 3.6. Omega-3 PUFAs Effects

During the study, compliance to the administered *ω*-3 PUFAs was optimal, with no side effects requiring the drop-out from the protocol treatment.

After *ω*-3 PUFAs supplementation, a significant reduction of clinical severity was observed in* MECP2*-RTT patients, as compared to the basal status before treatment population (CCSS −22.4%; *P* < 0.05). These data confirm our previous results in typical RTT [13, 14, 59]. A significant decrease in clinical severity (CCSS −37%; *P* < 0.05) was also observed in *ω*-3 PUFAs supplemented* CDKL5*-RTT population.

In supplemented* MECP2*-RTT patients, a significant decrease in TNF-*α*, IL-4, IL-17A, IL-37, IL-8, and RANTES plasma levels (*P* < 0.05) was evidenced, whereas significantly increased levels were observed for IFN-*γ*, IL-12p70, IL-6, IL-10, IL-13, IL-33, IL-22, IP-10, and I-TAC plasma levels (*P* < 0.05) as compared to unsupplemented* MECP2*-RTT patients. No significant changes were observed for IL-1*β*, IL-5, and TGF-*β*1 (Figures 1, 2, 3, and 4) [55–58].

When compared to unsupplemented* MECP2*-RTT patients, IL-4/IFN-*γ* and IL-6/IFN-*γ* ratios were decreased whereas the IL-10/IFN-*γ* ratio was increased (*P* < 0.05). As it concerns TNF-*α* based ratios, IL-5/TNF-*α* and IL-6/TNF-*α* were increased, whereas IL-4/TNF-*α* was reduced (*P* < 0.05) (Figure 5).

In supplemented* CDKL5*-RTT patients, a significant decrease in TNF-*α*, IL-10, IL-13, IL-8, and IP-10 plasma levels (*P* < 0.05) was evidenced, as compared to unsupplemented* CDKL5*-RTT patients, whereas significantly increased levels were evidenced for IL-4, IL-6, TGF-*β*1, and I-TAC (*P* < 0.05). No significant changes were observed for IFN-*γ*, IL-1*β*, IL-5, IL-17A, IL-37, and RANTES (Figures 1, 2, 3, and 4) [55–58]. The evaluation of circulating IL-12p70, IL-33, and IL-22 was not applicable due to insufficient sample volumes.

When compared to unsupplemented* CDKL5*-RTT patients, only IL-10/IFN-*γ* ratio was decreased whereas all ratios based on TNF-*α* were increased (*P* < 0.05) (Figure 5).

Interestingly, *ω*-3 PUFAs induced a similar hypersecretion in IL-6 associated with a similar depression in TNF-*α* and IL-8 circulating levels for both* MECP2*- and* CDKL5*-RTT.

In supplemented* MECP2*-RTT, all examined OS markers were significantly decreased (*P* < 0.05) and GSH/GSSG ratio increased (*P* < 0.05). Only IE-NPBI levels resulted as normalized, as compared to those of the control subjects (Figure 7).

In supplemented* CDKL5*-RTT, P-NPBI, IE-NPBI, and F_2_-IsoPs levels were significantly decreased as compared to those of unsupplemented* CDKL5*-RTT (*P* < 0.05). No differences were observed for F_4_-NeuroPs, F_2_-dihomo-IsoPs, and GSH/GSSG ratio (Figure 7).

Following 12 months of *ω*-3 PUFAs supplementation, ESR values were significantly reduced in* MeCP2*-RTT as compared to untreated* MECP2*-population (−43.5%) but remained elevated as compared to healthy subjects. In supplemented* CDKL5*-RTT patients, ESR values were decreased (−46.2%) and the values were similar to those of the control group (Figure 8).

Overall, *ω*-3 PUFAs appear to modulate Th1-related cytokines in a similar way in both* MECP2*-RTT and* CDKL5*-RTT. This counterbalance was mirrored by decreased OS markers levels and ESR values.

#### 3.6.1. Summary Effects of *ω*-3 PUFAs Supplementation on Circulating Cytokine Changes in* MECP2*- and* CDKL5*-RTT

In* MECP2*-RTT, a prolonged high-dosage strongly suppressed TNF-*α* production and regulated the Th1 cytokine pattern and T-reg lineage as well as Th2 with the exception of IL-4. As it concerns chemokines, *ω*-3 PUFAs effect seems to be regulatory with a single exception of a persistently inhibited RANTES secretion. IL-37 appears to be inhibited as the likely net result of anti-inflammatory action (Figure 6(c)).

As compared to the* MECP2*-RTT picture, the effects of *ω*-3 PUFAs in* CDKL5*-RTT appear to be more difficult to be interpreted in a univocal way. For Th1 lineage, *ω*-3 PUFAs modulate the examined cytokines in a similar way to that observed in* MECP2*-RTT. Paradoxical responses within the Th2 and T-reg lineages were observed. As far as chemokines are concerned, *ω*-3 PUFAs partially counteract baseline changes, with the exceptions of IP-10 and RANTES. On the other hand, IL-37 levels appear to be unchanged (i.e., elevated levels) (Figure 6(d)).

## 4. Discussion

Our findings indicate, for the first time, a complex cytokine dysregulation in RTT. In particular, in* MECP2*-RTT, a Th2-shifted balance was evidenced, whereas in* CDKL5*-RTT both Th1- and Th2-related (except for IL-4) cytokines were found to be upregulated.

Almost specular changes were observed regarding IL-22 and T-reg cytokine TGF-*β*1 between these disorders, decreased IL-22 and unchanged T-reg levels in* MECP2*-RTT* versus* increased IL-22 and T-reg levels in* CDKL5*-RTT.

The observed cytokine dysregulation was proportional to clinical severity, inflammatory status, and redox imbalance. Interestingly, the macrophage has been very recently reported as a key target cell for MeCP2 action in a mouse model of* Mecp2* loss-of-function [28]. Interestingly, several of the assayed cytokines, whose circulating levels were found to be abnormal in* MECP2*-RTT girls, included macrophage-related cytokines, such as TNF-*α*, IL-6, IL-12p70, IL-10, TGF-*β*1, IL-8, and RANTES [60].

Although the characteristic cytokine pattern in RTT appears to be inhomogeneous, it includes high plasma levels of TNF-*α* and IL-5. TNF-*α* is a proinflammatory cytokine and produced in response to inflammatory stimuli. Elevated levels of TNF-*α* have been reported in rheumatoid arthritis, ankylosing spondylitis, irritable bowel disease, and psoriasis [61]. IL-5, a Th2 cytokine, acts on eosinophils, basophils, mast cells, and B cells and elevated levels were found in allergy asthma and hypereosinophilic syndrome [62].

Actually, none of the clinical features observed in these associated pathologies are usually present in RTT, either* MECP2*- or* CDKL5*-related disorder.

Our data confirm the presence of a significant proinflammatory status in* MECP2*-RTT. For the first time, we observed an upregulation of both Th1- and Th2-related cytokines in CDKL5-RTT, which does not translate into increased inflammatory marker levels, likely due to a bulk increase in the anti-inflammatory IL-10 (about 21-fold), a key regulator cytokine of immune response [50].

The clinical translation for the observed changes remains to be elucidated. Nevertheless, cytokine dysregulation in* MECP2*-RTT appears to be associated with a proinflammatory status, as evidenced by raised ESR values and an acute phase protein response [24]. On the other hand, the final result in* CDKL5*-RTT remains unclear, given that ESR was found to be slightly higher than control values, although not statistically different. We have previously described in* MECP2*-RTT patients the presence of respiratory bronchiolitis-associated interstitial lung disease- (RB-ILD-) like features at the high-resolution CT (HRCT) images of the lungs in up to half of patients aged 10 years or more [63]. More recently, histologic evidence for an inflammatory lung disease has been reported by our group in a murine model of the disease [25]. Therefore, it is conceivable that the unexplained persistent inflammatory process in* MECP2*-RTT could be the possible result of an ineffective chemotaxis, at least in some anatomical districts. No information to this regard is available to date for* CDKL5*-RTT.

**Figure 9:**
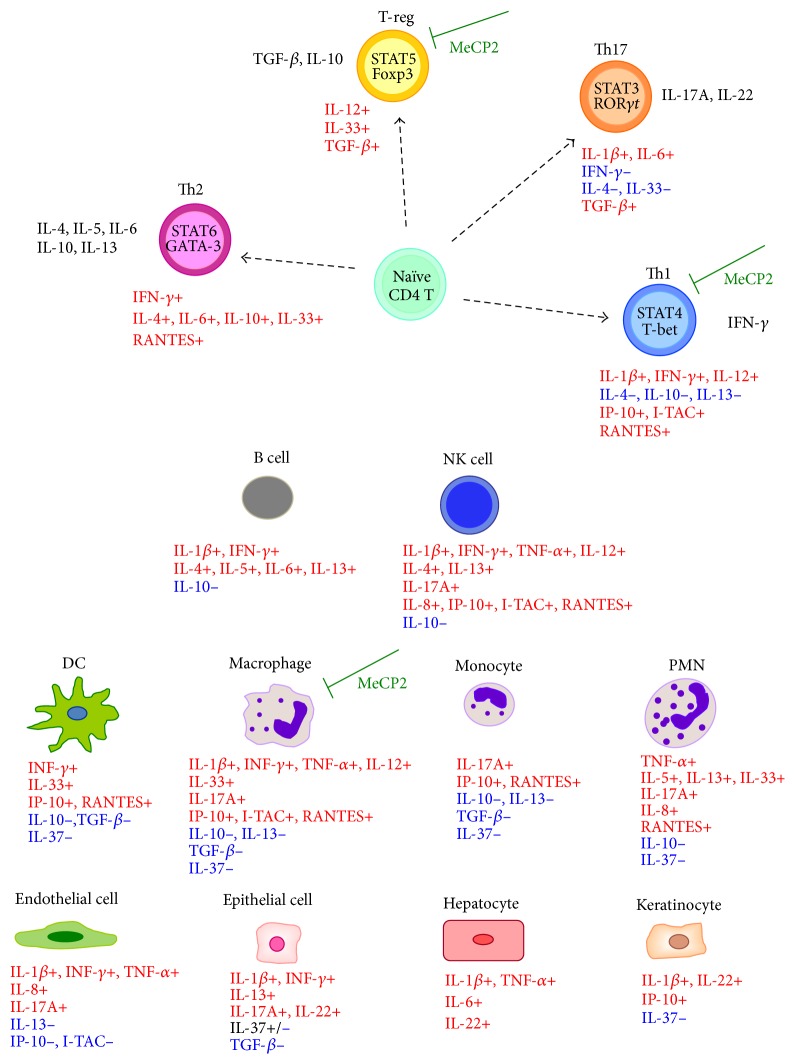
Schematic overview of the known effects of cytokines on immune and nonimmune cells. Blue font indicates anti-inflammatory effects. Red font indicates proinflammatory/activation effects. Relationships were reconstructed on the basis of literature [61, 62, 64, 66]. The modulatory effects of MeCP2 are referred to in [27, 28, 30]. Th1: T helper 1 cell; Th2: T helper 2 cell; Th17: T helper 17 cell; T-reg: T-regulatory cell, NK cell: natural killer cell; DC: dendritic cell; PMN: polymorphonuclear leukocyte.

Prior reports have suggested the occurrence of defective Th1 differentiation in RTT [30, 34]. Our findings in* MECP2*-RTT appear to be in line with this suggestion, given that levels of cytokines known to be produced or related to Th1 are generally reduced, with the only notable exception of TNF-*α* (Figure 9) [61, 64]. On the contrary, Th1 cytokines were found to be all increased in* CDKL5*-RTT. To date, no information concerning immunological response, inflammatory status, or defense against infections is available for this condition.

On the other hand, Th2-cytokines were found to be generally increased in RTT. In particular, a significant increase in IL-5, IL-6 was observed in* MECP2*-RTT whereas in* CDKL5*-RTT IL-5, IL-10, and IL-13 levels were increased. Thus the Th2-dependent response appears to be upregulated in RTT independently of the mutated gene. This observation is also in line with the hypothesis of a compensatory effect of immune response related to a defective Th1 differentiation [30, 34]. Possible clinical consequences of this Th2/Th1 shift imbalance in RTT could be a risk factor for autoimmunity, which is consistent with earlier reports on a link between MeCP2 and autoimmunity [41–43].

Although Fiumara et al. [31] did not find anti-neuronal and anti-myelin ganglioside as well as anti-nuclear, anti-striated muscle, and anti-smooth muscle autoantibodies, a recent report by Papini et al. [44] evidenced high levels of serum anti-N(Glc) autoantibodies in RTT.

The immune regulatory response appears to be also aberrant in RTT in that while the cytokines produced by T-reg cells (i.e., IL-10, TGF-*β*1) [50] were unchanged in* MECP2*-RTT, they appear to be increased in* CDKL5*-RTT. T-reg cells are critical in regulating the inflammatory process and Mecp2 has been recently shown to be a crucial player in determining T-reg resilience against inflammation [27]. The loss of control of the T-reg function is one of the known causes for the loss of the immune tolerance [65].

Interestingly, the behaviour of circulating IL-22 was the opposite in* MECP2*-RTT and in* CDKL5*-RTT. Although IL-22, a proinflammatory cytokine, is considered to be upregulated in chronic inflammatory diseases, its role seems to be depending on the biological context in that it can be able to favour the antimicrobial defense, regeneration, and protection against damage and induce acute phase reactants, as well as some chemokines [66]. Another proinflammatory cytokine, IL-17A, when compared to our control group, was found to be significantly increased in* MECP2*-RTT but significantly decreased in* CDKL5*-RTT. Likewise, when compared to our control group, levels of the anti-inflammatory cytokine IL-37 [49] were found to be significantly elevated in* MECP2*-RTT as the consequence of a compensatory mechanism.

Overall, the observed cytokine dysregulation does not appear to translate into a primary immunodeficiency, nor in a classical autoimmune disease [44].

Although the reasons behind to date observed cytokine dysregulation in RTT are unknown, possible explanations may include either a defective genetic/epigenetic control on target genes or an aberrant redox imbalance. Our findings, combined with published evidence, suggest that* Mecp2* loss-of-function mutations could lead to abnormal redox homeostasis via the Jak/STAT3 signaling pathway [67, 68]. Our demonstration of a redox imbalance in* MECP2*-RTT and* CDKL5*-RTT confirms and extends prior evidence of OS as a hallmark feature of RTT [16, 18]. Oxidation of polyunsaturated fatty acids, with consequent enhanced formation of IsoPs and NeuroPs [69, 70], is one of major features of OS. A key difference between* MECP2*-RTT and* CDKL5*-RTT appears to reside in the F_2_-dihomo-IsoPs behaviour, in that their levels are comparable to those of control group in* CDKL5*-RTT, whereas they are significantly increased in* MECP2*-RTT. To date, no clear explanation exists for these findings; it might reflect the clinical difference between two disorders. Clinical differences between the disorders may account for the observed redox pattern differences, in that* MECP2*-RTT can be considered a multisystemic disease, whereas* CDKL5*-RTT is essentially a myoclonic encephalopathy.

Therefore, a complex interplay exists between cytokines, redox homeostasis, and inflammatory status in RTT. Statistically, cytokine levels were found to explain a very consistent fraction of the observed variance for subclinical inflammation and redox abnormality, thus indicating that the aberrant immune response, as regulated by cytokine signalling, is intimately related to redox imbalance and both are likely responsible for modulating phenotype severity in RTT.

Likely, the more striking result of our study is the evidence of a persistent and unexplained hyper-TNF-*α* status in RTT. Hyperleptinemia in* MECP2*-RTT has been previously reported by Blardi et al. [71]. The findings of our study would allow a novel data interpretation from a different perspective. Leptin is able to modulate both innate and adaptive immune response [1]. Indeed, the overall leptin action in the immune system is that of a proinflammatory effect, activating proinflammatory cells, promoting Th1 responses, and mediating the production of the other proinflammatory cytokines, such as TNF-*α*, IL-2, or IL-6 [72]. Therefore, it is plausible that, in untreated* MECP2*-RTT girls, the hyper-TNF-*α* status might be triggered by hyperleptinemia.

A beneficial effect of *ω*-3 PUFAs in* MECP2*-RTT has been previously reported by our group on redox homeostasis [13, 14, 59, 73, 74], subclinical inflammation [75], fatty acid composition of erythrocyte membranes [76], and clinical severity [13, 14, 59, 73].

EPA and DHA, that is, the major *ω*-3 PUFAs contained in fish oil, are known to partly inhibit several aspects of inflammation, including leukocyte chemotaxis, adhesion molecule expression, production of eicosanoids, production of inflammatory cytokines, and T-helper 1 lymphocyte reactivity [77]. The critical interplay between inflammation and OS in the underlying mechanisms leading from gene mutation to disease expression [78] is further supported by the modulatory effect of *ω*-3 PUFAs on cytokine patterns in RTT. Our findings demonstrate that *ω*-3 PUFAs partially counterbalance cytokine changes, aberrant redox homeostasis, and proinflammatory status.

In particular, a consistent number of the investigated cytokines appear to be rescued following a 12-month high dosage supplementation. Target cytokines for *ω*-3 PUFAs in* MECP2*-RTT include TNF-*α*, IFN-*γ*, IL-4, IL-17A, TGF-*β*1, IL-22, IL-37, IL-8, IP-10, and I-TAC, whereas they include TNF-*α*, IL-4, IL-5, IL-13, IL-17A, IL-8, IP-10, and I-TAC in* CDKL5*-RTT. These data appear to further support and extend prior reports on the immunomodulatory effects of DHA and EPA in biological systems.

The present study indicates that RTT is associated with a subclinical immune dysregulation, as a likely consequence of a defective inflammation regulatory signaling system. This abnormal regulation of the inflammatory response appears to be an unrecognized hallmark feature of RTT, intimately related to OS imbalance and likely contributing to disease expression.

## Supplementary Material


*Relative changes (as fold variations) for inflammatory status, circulating cytokines, and redox status in MECP2- and CDKL5- mutated Rett patients*. In order to account for real changes of the examined variables, i.e., independently of absolute basal concentrations, we performed data analysis through a double standardization (i.e., basal values of untreated *MECP2*- and *CDKL5*-RTT patients vs. control values and treated *MECP2*- and *CDKL5*-RTT patients vs. untreated) (Supplementary Table 1 and Table 2). After standardization, some differences loosed weight in both ω -3 PUFAs supplemented *MECP2*- and *CDKL5*-RTT. In particular, changes in IL-10 in *MECP2*-RTT and changes in IL-6, TGF-b1, IL-37, IP-10, IL-5/ TNF-α ratio, IL-6/ TNF-α ratio, IL-10/ TNF-α ratio in CDKL5-RTT were found to be not significant. Likewise, other differences gained statistical significance as changes in IL-5, IL-6/IFN-g ratio, IL-4/ TNF-α ratio, IL-6/ TNF-α ratio in *MeCP2*-RTT and IFN-g, IL-1b, IL-5, IL-37, IL-4/ IFN-g ratio, F_4_-NeuroPs, and GSH/GSSG ratio in CDKL5-RTT.

## Figures and Tables

**Table 1:** Correlation coefficients for the relationships between clinical severity, inflammation, redox status, and circulating cytokine.

	Clinical severity (CCSS)	ESR	P-NBPI	IE-NPBI	F_2_-IsoPs	F_4_-NeuroPs	F_2_-dihomo-IsoPs	GSH/GSSG ratio
Clinical severity (CCSS)	—	0.328^**∗∗∗**^	0.279^**∗∗**^	0.290^**∗∗**^	0.461^**∗∗∗**^	0.492^**∗∗∗**^	0.409^**∗∗∗**^	−0.326^**∗∗∗**^
ESR	—	—	0.424^**∗∗∗**^	0.276^**∗∗**^	0.456^**∗∗∗**^	0.586^**∗∗∗**^	0.494^**∗∗∗**^	−0.397^**∗∗∗**^
TNF-*α*	0.456^**∗∗∗**^	0.438^**∗∗∗**^	0.713^**∗∗∗**^	0.661^**∗∗∗**^	0.678^**∗∗∗**^	0.749^**∗∗∗**^	0.634^**∗∗∗**^	−0.310^**∗∗∗**^
IFN-*γ*	−0.305^**∗∗∗**^	−0.252^**∗∗**^	**−**0.091	**−**0.154	**−**0.088	−0.362^**∗∗∗**^	−0.391^**∗∗∗**^	**−**0.066
IL-1*β*	−0.231^**∗∗**^	**−**0.117	0.218^**∗∗**^	0.091	0.145	**−**0.101	−0.176^**∗**^	−0.158^**∗**^
IL-12p70	−0.399^**∗∗∗**^	−0.315^**∗∗∗**^	−0.243^**∗∗**^	−0.302^**∗∗**^	−0.189^**∗**^	−0.571^**∗∗∗**^	−0.624^**∗∗∗**^	−0.213^**∗∗**^
IL-4	0.419^**∗∗∗**^	0.520^**∗∗∗**^	0.483^**∗∗∗**^	0.456	0.508^**∗∗∗**^	0.816^**∗∗∗**^	0.787^**∗∗∗**^	**−**0.233
IL-5	0.006	0.024	0.144	0.022	0.233^**∗∗**^	0.052	**−**0.030	−0.372^**∗∗∗**^
IL-6	0.002	0.138	**−**0.042	**−**0.063	0.052	0.105	0.012	−0.480^**∗∗∗**^
IL-10	−0.219^**∗**^	**−**0.108	0.218^**∗∗**^	0.087	0.214^**∗∗**^	−0.212^**∗∗∗**^	−0.288^**∗∗**^	−0.250^**∗∗**^
IL-13	**−**0.068	**−**0.071	**−**0.033	**−**0.066	0.073	−0.173^**∗**^	−0.247^**∗∗**^	−0.316^**∗∗∗**^
IL-33	0.080	0.190^**∗**^	0.197^**∗**^	0.113	0.268^**∗∗∗**^	0.286^**∗∗∗**^	0.149	−0.582^**∗∗∗**^
IL-17A	0.446^**∗∗∗**^	0.555^**∗∗∗**^	0.431^**∗∗∗**^	0.392^**∗∗∗**^	0.382^**∗∗**^	0.667^**∗∗∗**^	0.675^**∗∗∗**^	−0.222^**∗∗**^
IL-22	−0.379^**∗∗∗**^	−0.427^**∗∗∗**^	−0.549^**∗∗**^	−0.548^**∗∗∗**^	−0.558^**∗∗∗**^	−0.817^**∗∗∗**^	−0.821^**∗∗∗**^	0.283^**∗∗**^
TGF-*β*1	−0.246^**∗∗**^	−0.309^**∗∗∗**^	0.099	**−**0.043	**−**0.074	−0.218^**∗∗**^	−0.235^**∗∗**^	0.010
IL-37	0.197^**∗**^	0.431^**∗∗∗**^	0.525^**∗∗**^	0.409^**∗∗**^	0.625^**∗∗∗**^	0.625^**∗∗∗**^	0.499^**∗∗**^	−0.556^**∗∗∗**^
IL-8	0.173	0.180^**∗**^	0.321^**∗∗**^	0.322^**∗∗**^	0.392^**∗∗∗**^	0.361^**∗∗∗**^	0.358^**∗∗**^	**−**0.151
IP-10	**−**0.110	−0.191^**∗**^	−0.397^**∗∗**^	−0.259^**∗∗**^	−0.365^**∗∗**^	−0.377^**∗∗**^	−0.392^**∗∗**^	0.443^**∗∗∗**^
I-TAC	−0.189^**∗∗**^	−0.407^**∗∗∗**^	−0.664^**∗∗∗**^	−0.567^**∗∗∗**^	−0.697^**∗∗∗**^	−0.582^**∗∗∗**^	−0.502^**∗∗∗**^	−0.447^**∗∗∗**^
RANTES	0.012	−0.284^**∗∗∗**^	**−**0.134	**−**0.102	**−**0.249	−0.278^**∗∗**^	0.176^**∗**^	0.541^**∗∗∗**^
IL-4/IFN-*γ*	0.302^**∗∗**^	0.336^**∗∗∗**^	0.456^**∗∗∗**^	0.393^**∗∗∗**^	0.516^**∗∗∗**^	0.817^**∗∗∗**^	0.687^**∗∗∗**^	−0.209^**∗∗**^
IL-5/IFN-*γ*	0.242^**∗∗**^	0.499^**∗∗∗**^	0.266^**∗∗**^	0.221^**∗∗**^	0.272^**∗∗**^	0.449^**∗∗∗**^	0.443^**∗∗∗**^	**−**0.133
IL-6/IFN-*γ*	0.174	0.321^**∗∗∗**^	0.164^**∗**^	0.128	0.220^**∗∗**^	0.367^**∗∗∗**^	0.290^**∗∗**^	−0.411^**∗∗∗**^
IL-10/IFN-*γ*	**−**0.141	**−**0.032	0.189^**∗**^	0.098	0.174^**∗**^	**−**0.132	−0.197^**∗**^	−0.229^**∗∗**^
IL-4/TNF-*α*	−0.235^**∗∗**^	−0.166^**∗**^	**−**0.115	−0.179^**∗∗**^	0.470^**∗∗∗**^	0.704^**∗∗∗**^	**−**0.117	0.008
IL-5/TNF-*α*	−0.302^**∗∗∗**^	−0.162^**∗**^	**−**0.134	−0.203^**∗∗**^	**−**0.149	−0.214^**∗∗∗**^	−0.175^**∗**^	**−**0.005
IL-6/TNF-*α*	**−**0.124	**−**0.098	**−**0.113	−0.165^**∗**^	**−**0.103	**−**0.149	**−**0.127	**−**0.111
IL-10/TNF-*α*	−0.228^**∗∗**^	**−**0.065	0.023	**−**0.055	0.059	**−**0.109	**−**0.137	**−**0.127

Data are expressed as correlation coefficients. Bold characters indicate statistically significant associations. Asterisks denote *P* values: ^*∗*^ *P* < 0.05; ^*∗∗*^0.05 < *P* < 0.001; ^*∗∗∗*^ *P* < 0.0001. CCSS: compressed clinical severity score [46]; ESR: erythrocyte sedimentation rate; P-NPBI: plasma non-protein-bound iron; F_2_-IsoPs: plasma F_2_-isoprostanes; F_2_-dihomo-IsoPs: plasma F_2_-dihomo-isoprostanes; F_4_-NeuroPs: plasma F_4_-neuroprostanes; IE-NBPI: intraerythrocyte non-protein-bound iron; GSH: reduced glutathione; GSSG: oxidized glutathione.

**Table 2:** Relationship between clinical severity, redox status, subclinical inflammation, and circulating cytokines for the whole population: results of a stepwise multiple regression analysis.

Dependent variable	Final stepwise model			*R* ^2^	*R* ^2^ adjusted	Multiple correlation coefficient	Residual SD	*F*-ratio	Significance of *P* value
	Selected predictor variables	*R* partial	*P* value						
Clinical severity (CCSS) [46]	(+) TNF-*α*	0.406	<0.0001	0.490	0.476	0.699	2.66	35.72	**<0.001**
	(+) IL-17A	0.235	0.0037						
	(+) IL-4	0.362	<0.0001						
	(−) IL-12p70	0.581	<0.0001						

Clinical severity (CCSS) [46]	(−) GSH/GSSG	0.779	<0.0001	0.809	0.805	0.899	1.59	220.39	**<0.001**
	(+) F_4_-NeuroPs	0.235	0.0029						
	(+) F_2_-IsoPs	0.184	0.0205						

ESR	(+) IL-17A	0.319	<0.0001	0.304	0.292	0.552	10.84	25.38	**<0.001**
	(+) IL-4/IFN-*γ*	0.475	0.0009						

P-NPBI	(+) TNF-*α*	0.532	<0.0001	0.563	0.556	0.750	0.18	74.79	**<0.001**
	(−) I-TAC	0.358	0.0001						

F_2_-IsoPs	(+) TNF-*α*	0.383	<0.0001	0.581	0.570	0.762	12.93	53.23	**<0.001**
	(+) IL-37	0.285	0.0018						
	(−) I-TAC	0.434	<0.0001						

F_2_-dihomo-IsoPs	(−) IL-22	0.596	<0.0001	0.711	0.704	0.843	9.95	94.52	**<0.001**
	(+) IL-4/IFN-*γ*	0.260	0.0047						
	(+) IL-17A	0.194	0.0359						

F_4_-NeuroPs	(−) IL-22	0.553	<0.0001	0.874	0.870	0.935	2.26	197.65	**<0.001**
	(+) IL-4/IFN-*γ*	0.247	0.0074						
	(+) IL-37	0.201	0.0235						
	(−) IL-12p70	0.606	<0.0001						

IE-NPBI	(+) TNF-*α*	0.493	<0.0001	0.440	0.431	0.664	0.32	48.02	**<0.001**
	(−) I-TAC	0.221	0.0136						

GSH/GSSG ratio	(+) IL-22	0.678	<0.0001	0.804	0.797	0.897	1.89	117.05	**<0.001**
	(−) IL-37	0.247	0.0076						
	(−) RANTES	0.476	<0.0001						
	(+) I-TAC	0.684	<0.0001						

Plus and minus signs in brackets indicate positive or negative associations. CCSS: compressed clinical severity score [46]; ESR: erythrocyte sedimentation rate; P-NPBI: plasma non-protein-bound iron; F_2_-IsoPs: plasma F_2_-isoprostanes; F_2_-dihomo-IsoPs: plasma F_2_-dihomo-isoprostanes; F_4_-NeuroPs: plasma F_4_-neuroprostanes; IE-NBPI: intraerythrocyte non-protein-bound iron; GSH: reduced glutathione; GSSG: oxidized glutathione.
