# Supplementary material for: Cytokine Dysregulation in MECP2- and CDKL5-Related Rett Syndrome: Relationships with Aberrant Redox Homeostasis, Inflammation, and ω-3 PUFAs
Source: Oxid Med Cell Longev. 2015 Jul 8;2015:421624. doi: 10.1155/2015/421624 (PMC4510261; doi:10.1155/2015/421624)
Supplement: Supplementary file 1 — Relative changes (as fold variations) for inflammatory status, circulating cytokines, and redox status in MECP2- and CDKL5- mutated Rett patients. In order to account for real changes of the examined variables, i.e., independently of absolute basal concentrations, we performed data analysis through a double standardization (i.e., basal values of untreated MECP2- and CDKL5-RTT patients vs. control values and treated MECP2- and CDKL5-RTT patients vs. untreated) (Supplementary Table 1 and Table 2). After standardization, some differences loosed weight in both ω -3 PUFAs supplemented MECP2- and CDKL5-RTT. In particular, changes in IL-10 in MECP2-RTT and changes in IL-6, TGF-b1, IL-37, IP-10, IL-5/ TNF-α ratio, IL-6/ TNF-α ratio, IL-10/ TNF-α ratio in CDKL5-RTT were found to be not significant. Likewise, other differences gained statistical significance as changes in IL-5, IL-6/IFN-g ratio, IL-4/ TNF-α ratio, IL-6/ TNF-α ratio in MeCP2-RTT and IFN-g, IL-1b, IL-5, IL-37, IL-4/ IFN-g ratio, F4-NeuroPs, and GSH/GSSG ratio in CDKL5-RTT. [file 421624.f1.pdf]

**Supplementary Table 1.** Changes (folds *vs.* controls) for inflammatory status, circulating cytokines, and redox status in *MECP2*- and *CDKL5*- mutated Rett patients.

| | ***MECP2*-mutated Rett patients** | ***CDKL5*-mutated Rett patients** |
|---|---|---|
| *Inflammatory status* | | |
| ESR | **3.31 ± 1.77** | 1.52 ± 0.48 |
| *Circulating cytokines* | | |
| TNF-α | **7.51 ± 2.32** | **4.56 ± 1.73** |
| IFN-γ | **0.63 ± 0.22** | **1.80 ± 1.12** |
| IL-1β | 0.96 ± 0.41 | **3.41 ± 1.45** |
| IL-12p70 | 0.00066 ±0.00037 | 3.15 ± 0.54 |
| IL-4 | **3.00 ± 0.38** | **0.69 ± 0.49** |
| IL-5 | **1.05 ± 0.09** | **1.14 ± 0.04** |
| IL-6 | **2.82 ± 1.00** | **1.75 ± 1.83** |
| IL-10 | 0.70 ± 0.95 | **21.22 ± 2.79** |
| IL-13 | 1.17 ± 0.79 | **4.56 ± 1.60** |
| IL-33 | **6.28 ± 1.94** | **4.57 ± 1.72** |
| IL-17A | **3.00 ± 1.12** | **0.30 ± 0.25** |
| IL-22 | **0.46 ± 0.13** | 1.10 ± 0.16 |
| TGF-β1 | **0.94 ± 0.12** | **1.14 ± 0.03** |
| IL-37 | **4.25 ± 1.33** | **3.16 ± 1.61** |
| IL-8 | **1.63 ± 0.62** | **1.40 ± 0.72** |
| IP-10 | **0.69 ± 0.12** | **0.71 ± 0.12** |
| I-TAC | **0.47 ± 0.11** | **0.40 ± 0.14** |
| RANTES | **0.86 ± 0.09** | 0.95 ± 0.06 |
| *Th2/Th1 ratio* | | |
| IL-4 / IFN-γ | **3.99 ± 2.14** | 0.60 ± 0.37 |
| IL-5 / IFN-γ | **1.79 ± 0.72** | 0.81 ± 0.50 |

| | | |
|---|---|---|
| IL-6 / IFN-γ | **4.87 ± 1.59** | 1.28 ± 1.14 |
| IL-10 / IFN-γ | 1.10 ± 1.04 | **8.13 ± 5.64** |
| IL-4 / TNF-α | 0.26 ± 1.14 | 0.27 ± 0.25 |
| IL-5 / TNF-α | 0.14 ± 0.08 | 1.17 ± 1.15 |
| IL-6 / TNF-α | 0.10 ± 0.06 | 3.17 ± 3.06 |
| IL-10 / TNF-α | 0.12 ± 0.09 | 20.96 ± 6.85 |
| *OS markers* | | |
| P-NPBI | **2.57 ± 0.69** | **2.53 ± 0.31** |
| IE-NPBI | **2.08 ± 0.82** | **1.89 ± 0.38** |
| $F_2$-IsoPs | **3.58 ± 0.82** | **3.44 ± 0.68** |
| $F_4$-NeuroPs | **17.47 ± 2.12** | **4.64 ± 1.02** |
| $F_2$-dihomo-IsoPs | **34.67 ± 3.56** | 0.73 ± 0.51 |
| GSH/GSSG ratio | **0.051 ± 0.002** | **0.070 ± 0.005** |

Data are expressed as folds *vs.* healthy subjects values and indicated as means ± standard deviations. Value < 1 indicates a decrease in the examined variables (*i.e.*, inflammatory markers, cytokine production, OS markers) whereas value > 1 indicates an increase in the examined variables. Bold characters indicate statistical significance ($P < 0.05$). ESR: erythrocyte sedimentation rate; P-NPBI: plasma non-protein-bound iron; $F_2$-IsoPs: plasma $F_2$-isoprostanes; $F_2$-dihomo-IsoPs: plasma $F_2$-dihomo-isoprostanes; $F_4$-NeuroPs: $F_4$-neuroprostanes; GSH: reduced glutathione; GSSG: oxidized glutathione.

**Supplementary Table 2.** Changes for clinical severity, inflammatory status, circulating cytokines, and redox status in *MECP2*- and *CDKL5*- mutated Rett patients after ω-3 PUFAs supplementation.

| | ***MECP2*-mutated Rett patients** | ***CDKL5*-mutated Rett patients** |
|---|---|---|
| *Clinical severity* | | |
| *CCSS* [46] | **0.77 ± 0.4** | **0.63 ± 0.2** |
| *Inflammatory status* | | |
| ESR | **0.57 ± 0.24** | 0.54 ± 0.39 |
| *Circulating cytokines* | | |
| TNF-α | **0.072 ± 0.12** | **0.00013 ± 0.00001** |
| IFN-γ | **1.81 ± 0.53** | **0.99 ± 0.30** |
| IL-1β | 1.10 ± 0.55 | **0.97 ± 0.13** |
| IL-12p70 | **277 ± 8.58** | N.A. |
| IL-4 | **0.41 ± 0.08** | **2.14 ± 0.45** |
| IL-5 | **1.02 ± 0.05** | **0.94 ± 0.11** |
| IL-6 | **1.46 ± 0.75** | 2.36 ± 0.95 |
| IL-10 | 3.69 ± 3.0 | **0.49 ± 0.22** |
| IL-13 | **2.93 ± 1.87** | **0.13 ± 0.10** |
| IL-33 | **1.23 ± 0.63** | N.A. |
| IL-17A | **0.39 ± 0.23** | 0.004 ± 0.009 |
| IL-22 | **2.19 ± 0.31** | N.A. |
| TGF-β1 | **1.01 ± 0.08** | 1.19 ± 0.01 |
| IL-37 | **0.60 ± 0.23** | **1.02 ± 0.01** |
| IL-8 | **0.64 ± 0.38** | **0.0037 ± 0.0004** |
| IP-10 | **1.25 ± 0.36** | 0.44 ± 0.15 |
| I-TAC | **1.93 ± 0.51** | **2.03 ± 0.94** |
| RANTES | **0.94 ± 0.09** | 1.05 ± 0.02 |

| | | |
|---|---|---|
| *Th2/Th1 ratio* | | |
| IL-4 / IFN-γ | **0.27 ± 0.18** | **2.16 ± 1.17** |
| IL-5 / IFN-γ | **0.60 ± 0.40** | 0.93 ± 0.62 |
| IL-6 / IFN-γ | **0.88 ± 0.69** | 1.96 ± 1.20 |
| IL-10 / IFN-γ | **2.15 ± 2.10** | **0.43 ± 0.27** |
| IL-4 / TNF-α | **3.12 ± 2.49** | **6.0 ± 1.26** |
| IL-5 / TNF-α | **8.23 ± 3.99** | 4.65 ± 0.55 |
| IL-6 / TNF-α | **45.73 ± 11.2** | 6.32 ± 2.55 |
| IL-10 / TNF-α | 39.39 ± 11.3 | 1.83 ± 0.80 |
| *OS markers* | | |
| P-NPBI | **0.52 ± 0.08** | **0.66 ± 0.01** |
| IE-NPBI | **0.54 ± 0.07** | **0.47 ± 0.03** |
| $F_2$-IsoPs | **0.51 ± 0.14** | **0.45 ± 0.08** |
| $F_4$-NeuroPs | **0.29 ± 0.08** | **1.05 ± 0.06** |
| $F_2$-dihomo-IsoPs | **0.13 ± 0.12** | 6.62 ± 3.49 |
| GSH/GSSG ratio | **2.17 ± 0.09** | **0.86 ± 0.55** |

Data are expressed as folds *vs.* basal levels (*i.e.*, untreated *MECP2*- and *CDKL5*-mutated Rett patients) and indicated as means ± standard deviations. Value < 1 indicates a decrease in the examined variables (*i.e.*, inflammatory markers, cytokine production, OS markers) whereas value > 1 indicates an increase in the examined variables. Bold characters indicate statistical significance ($P < 0.05$). CCSS: compressed clinical severity score [46]; ESR: erythrocyte sedimentation rate; P-NPBI: plasma non-protein-bound iron; $F_2$-IsoPs, plasma $F_2$-isoprostanes; $F_2$-dihomo-IsoPs: plasma $F_2$-dihomo-isoprostanes; $F_4$-NeuroPs: $F_4$-neuroprostanes; GSH: reduced glutathione; GSSG: oxidized glutathione; N.A.: not applicable.
